# Quality of life and voice outcome of patients treated with transoral CO_2_ laser microsurgery for early glottic carcinoma (T1–T2): a 2-year follow-up study

**DOI:** 10.1007/s00405-019-05348-1

**Published:** 2019-02-27

**Authors:** Martine Hendriksma, Yda van Loon, W. Martin C. Klop, Marieke M. Hakkesteegt, Bas J. Heijnen, Ibtissam el Hasnaoui, Martin de Jong, Ton P. M. Langeveld, Peter Paul G. van Benthem, Robert J. Baatenburg de Jong, Elisabeth V. Sjögren

**Affiliations:** 1 Department of Otorhinolaryngology, Head and Neck Surgery, Leiden University Medical Center, Leiden, The Netherlands; 2 Department of Head and Neck Surgery, Netherlands Cancer Institute/Antoni van Leeuwenhoek, Amsterdam, The Netherlands; 3 Department of Otorhinolaryngology, Head and Neck Surgery, Erasmus MC Cancer Institute, Rotterdam, The Netherlands; 4 Department of Radiotherapy, Leiden University Medical Center, Leiden, The Netherlands

**Keywords:** Early glottic carcinoma, TLM, Laser surgery, Quality of life, Voice outcome, Questionnaire

## Abstract

**Purpose:**

Longitudinal studies in laryngeal cancer can provide clinicians information about short-term and long-term functional outcomes, like quality of life (QoL) and voice outcome. This information is important when counseling patients or choosing a primary treatment modality. The present study assessed long-term (2 years) QoL and voice outcome in patients with extended T1 and limited T2 glottic carcinoma treated with transoral CO_2_ laser microsurgery (TLM) (unilateral type III or bilateral type II resections).

**Methods:**

Three questionnaires were administered: the Voice Handicap Index (VHI), the European Organization for Research and Treatment of Cancer (EORTC) QoL questionnaire (QLQ)-C30, the EORTC QLQ-HN35. A perceptual voice evaluation at six different time points was conducted: preoperatively, and postoperatively at 6 weeks, 3 months, 6 months, 1 year, and 2 years. Fluctuations over time were investigated.

**Results:**

Sixty-one patients were included in the analysis. Patients reported high-level functioning and low symptom scores 2 years postoperatively. Gender significantly affected the VHI scores at 2 years (mean VHI scores: female 8.7 vs. male, 23.9; *p* = 0.023). The major improvement in VHI scores was observed within the first 6 months. The tumor stage (T1a, T1b, and T2) significantly impacted the grade (mean scores at 2 years: 1.0, 1.9, and 1.7; *p* = 0.001). These scores stabilized at 6 months.

**Conclusions:**

Patients show good long-term QoL with low symptom scores, a low voice handicap, and mild to moderate dysphonia, 2 years postoperatively. Scores stabilize at 6 months and provide a clear indication of status at 1 and 2 years.

## Introduction

Early glottic carcinoma (Tis-T2) can be treated effectively with radiotherapy or transoral CO_2_ laser microsurgery (TLM). According to the Dutch Guidelines for laryngeal carcinoma, TLM is the advocated treatment for superficial midcord T1a glottic carcinoma, and radiotherapy is indicated for more extended T1 and T2 glottic carcinomas [1]. Studies have shown that both therapies provided good, comparable oncological results [2–4], but some studies show superior laryngeal preservation after TLM [5–8]. There is less data on the functional outcomes of these treatment modalities, such as quality of life (QoL) and voice outcome, particularly in patients with T2 glottic carcinoma [7]. The lack of these data often prohibits adequate comparisons of modalities in patient counseling.

Although oncological results play a highly significant role in selecting the treatment modality, functional outcomes are also important when determining the patient’s treatment preferences. Each treatment modality has different side effects, and patients may have different preferences regarding the trade-offs. Therefore, treatment decisions for early glottic carcinoma should be based on both oncological and functional outcomes including patients’ preferences.

Several studies have investigated QoL in patients with early glottic carcinoma after treatment with radiotherapy or TLM [9–13]. Most have reported good postoperative QoL scores that were either the same or better than preoperative scores. A questionnaire that is often used and has a well-proven method to measure QoL in cancer patients is the European Organization for Research and Treatment of Cancer (EORTC) QoL questionnaire (QLQ) C30, which is a general questionnaire. This can be complemented with the specific head and neck cancer module, the EORTC QLQ-HN35. Both questionnaires ask the patient to rate their problems associated with their tumor and subsequent treatment and to reflect on their QoL. Voice outcome has also been studied in early glottic carcinoma after treatment with either radiotherapy or TLM. These studies showed that voice outcome improved significantly postoperatively after the treatment of Tis-T1a tumors (radiotherapy and TLM) or after limited resections (types I–II) (TLM) [12, 14, 15].

When the voice changes, it often affects patient’s self-perception, as well as how others perceive their voice. Although many acoustic and aerodynamic parameters can be determined, these perceptive changes, such as dysphonia in the form of hoarseness or breathiness and an increase in vocal effort, are often the most fundamental to the patients and their surroundings. Therefore, measures of voice outcome that are often used in the clinical setting are self-assessment tools such as the Voice Handicap Index (VHI) and perceptual evaluation tools, such as the GRBAS rating scale.

Both QoL and voice outcomes may vary, mainly depending on the timing of the evaluation [16]. Most prospective studies have reported preoperative and 3- to 12-month postoperative functional outcomes; in contrast, cross-sectional studies have only reported postoperative results, with large variations in time frames. The advantage of longitudinal studies is that they can assess changes over time and determine when a stable condition is achieved. This information can support clinicians in counseling patients about their long-term expectations, an essential component of a well-informed treatment decision. In light of these findings, and due to the lack of long-term functional outcome data, the present study aimed to (1) assess long-term (2-year) results of QoL and voice outcome in patients treated with TLM for extended T1 and limited T2 glottic tumors and (2) investigate fluctuations over time, based on prospectively collected data.

## Methods and materials

### Patients

From December 2009 to March 2015, this non-randomized, prospective, longitudinal outcome study was conducted at the University Cancer Center, Leiden, The Hague, the Erasmus Medical Center, and The Netherlands Cancer Institute/Antoni van Leeuwenhoek Hospital in The Netherlands. Included patients were those with extended T1N0 and limited T2N0 glottic carcinomas, which would require a unilateral transmuscular resection [European Laryngological Society (ELS) classification [17] type III] or a bilateral subligamental resection (type II), if treated surgically. All patients with lesions that met these criteria were offered a treatment choice between TLM and radiotherapy. Patients made their choice after comprehensive counseling (described elsewhere in detail by van Loon et al. [18]). After stroboscopy, the definite tumor stage was determined endoscopically under general anesthesia. Patients that met the inclusion criteria after endoscopy were enrolled in the study at that time. In case of T1b tumors, some procedures were staged to prevent web formation. The QoL and voice outcome were assessed with patient self-report questionnaires and perceptual voice analyses conducted at various time points during follow-up. The study was approved by the local Medical Ethics Committees at all three hospitals. Informed consent was obtained from all patients before inclusion into the study.

### Questionnaires

We implemented three self-administered, validated questionnaires: the VHI-30 [19], the EORTC QLQ-C30 version 3 [20], and the EORTC QLQ-HN35 [21]. Each was assessed at six different time points: preoperatively, and postoperatively, at 6 weeks, 3 months, 6 months, 1 year, and 2 years. Patients were asked to complete the questionnaires unaided during their visit to the outpatient clinic.

#### Voice Handicap Index

The Dutch version of the VHI is a validated 30-item questionnaire. It measures the psychosocial effects of voice impairments in daily life. Patients score each item by selecting a response from a five-point Likert scale, which ranges from 0 to 4 (0 = never, 4 = always). The sum of scores results in a total VHI score, which ranges from 0 to 120. A higher score indicates a worse voice-related outcome [19, 22]. A difference of ten points or more has been shown to be clinically relevant [23].

#### EORTC QLQ-C30

The EORTC-QLQ-C30 evaluates health-related QoL for the general population of patients with cancer. This questionnaire comprises 30 questions that address patient function and symptomatology over the preceding week. The questionnaire includes a global health status scale, five functional scales (physical, role, emotional, cognitive, and social), three multi-item symptom scales (fatigue, pain, and nausea and vomiting), and six single items that assess additional symptoms in patients with cancer (dyspnea, insomnia, appetite loss, constipation, diarrhea, and financial difficulties). Patients score each item by selecting a response from a four-point Likert scale from 1 (not at all) to 4 (very much), except for the global health status, which is scored from 1 (very poor) to 7 (excellent). These scores are transformed to a scale of 0–100. A higher score represents a higher (better) level of functioning or a higher (worse) level of symptoms [20]. A difference of ten points has been shown to be clinically relevant [24, 25].

#### EORTC QLQ-HN35

The EORTC QLQ-HN35 evaluates health-related QoL for patients with head and neck cancer. It is often used to complement the EORTC QLQ-C30. This questionnaire contains 35 questions that address symptoms and side effects of treatment, social function, and body image/sexuality. The questionnaire incorporates 7 multi-item scales: pain, swallowing, senses (taste and smell), speech, social eating, social contact, and sexuality; and 11 single items: teeth, opening mouth, dry mouth, sticky saliva, coughing, feeling ill, use of analgesics, nutritional supplements, feeding tube, weight loss, and weight gain. Each item is evaluated by selecting a response from a four-point Likert scale, the same as the scales used for the EORTC QLQ-C30. The final scores are transformed to a scale of 0–100. A higher score represents more severe problems or symptoms [21, 26]. A change of ten points has been shown to be clinically relevant [26].

### Perceptual evaluation and voice recording

Perceptual evaluation was performed with the GRBAS rating scale on a 30-s running speech sample. Recordings were acquired at a sampling frequency of 44.1 kHz with a dual microphone headset recorder (Alphatron Medical Systems) and a Beyer dynamic microphone, in a noise-free environment. The speech sample consisted of a standard, phonetically balanced Dutch text, “80 dappere fietsers” [80 brave cyclists]. The GRBAS rating scale consisted of five scales (grade, roughness, breathiness, asthenia, and strain), of which only the grade of dysphonia was rated because it reflects the overall degree of hoarseness or the severity of the voice abnormality [27]. Each sample was scored on a scale of 0 (normal voice) to 3 (severe dysphonic voice), and a higher score represented a more dysphonic voice [28]. A panel of four experienced listeners consisting of three speech–language pathologist and one ENT surgeon/laryngologist, all specialized in both oncological and benign voice pathology and treatment (B.J.H., M.M.H., V.A.H. vdK., E.V.S.),and blinded to all data scored the grade of dysphonia. In those cases where the experts rated the voice differently, consensus was reached through re-evaluation of the speech sample and discussion. The interrater reliability of our experts was of 0.91 (95% CI 0.89–0.93).

### Statistical methods

Statistical analyses were performed with SPSS version 23.0 (IBM Corp, Armonk, NY, USA). Assumptions of normality were assessed. The effect of time on the different questionnaires was assessed with the linear mixed model analysis; the model was adjusted for four possible confounders: gender, tumor stage, type of resection (unilateral type III vs. bilateral type II), and involvement of the anterior commissure (AC; no involvement vs. unilateral or bilateral involvement). Additionally, the least significant difference (LSD) post hoc test was used to adjust for multiple comparisons. The linear mixed model method was chosen, since it applies a correction for missing data. This correction is based on the observed data, and it uses all available information, without the need to censure an entire set of patient data, when one or more data points are missing or the need for imputation of measurements [29]. A *p* value of 0.05 was considered statistically significant.

## Results

### Patients

**Fig. 1:**
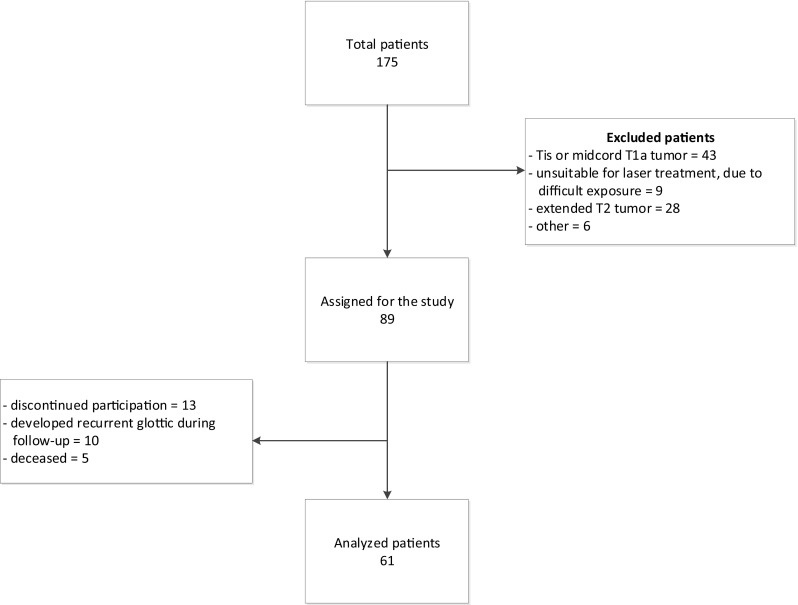
Flow diagram of patient selection for the study

One hundred and seventy-five patients with suspected or proven extended T1 and limited T2 glottic tumors were identified as candidates for the study. Of these, 89 were suitable for inclusion, based on endoscopy. Of these, 13 patients were lost to follow-up or discontinued participation in the first 3 months of the study. During the 2 years of follow-up, five patients died, due to unrelated causes, and ten patients developed recurrent disease. Thus, the final cohort comprised 61 patients for analysis (Fig. 1). The baseline characteristics of these patients are presented in Table 1.

**Table 1:** Baseline characteristics

Characteristics	Number of patients (%) Total = 61 (100%)
Mean age at surgery, years (SD)	67.6 (8.90)
Gender	
Male	51 (83.6)
Female	10 (16.4)
Tumor stage	
T1a	29 (47.5)
T1b	19 (31.1)
T2	13 (21.3)
Resection (ELS classification)	
Type III	38 (62.3)
Type II bilateral	23 (37.7)
AC involvement	
No	12 (19.7)
Unilateral	26 (42.6)
Bilateral	23 (37.7)

*AC* anterior commissure, *ELS* European Laryngological Society, *SD* standard deviation

### Voice Handicap Index

The mean VHI score improved significantly over time, ranging from 30.5 preoperatively to 21.8 at 2 years (∆8.7, *p* = 0.003,). However, according to our definition, this improvement did not qualify as clinically relevant. The major improvement in the VHI score occurred within the first 6 months (∆7.2). Thereafter, only small additional improvements were noted between 6 months and the 2-year follow-up. Gender was the only variable that significantly affected the VHI score; the difference in mean VHI scores was 11.6 points (*p* = 0.023) between male and female patients. The difference in mean VHI score per time point between male and female patients was 7.8 points (*p* = 0.204) preoperative, 14.6 points (*p* = 0.025) at 6 weeks, 11.0 points (*p* = 0.090) at 3 months, 15.4 points at 6 months (*p* = 0.018), 5.9 points (*p* = 0.346) at 1 year, and 15.1 (*p* = 0.039) points at 2 years. At 2 years, females showed a lower (normalized) VHI score and a larger improvement (∆15.3) than males. Additionally, unlike the improvement observed in male patients, the improvement in the mean VHI score in female patients was clinically relevant (Table 2).

**Table 2:** Voice Handicap Index and perceptual evaluation results

Groups	Preoperative Mean	6 weeks Mean (∆)	3 months Mean (∆)	6 months Mean (∆)	1 year Mean (∆)	2 years Mean (∆)	*p* value
Voice Handicap Index							
Overall (*n* = 61)	30.5	30.7 (0.23)	27.0 (− 3.5)	23.3 (− 7.2)	23.8 (− 6.7)	21.8 (− 8.7)	0.003
Male (*n* = 51)	31.8	33.1 (1.3)	28.8 (− 3.0)	25.7 (− 6.0)	24.8 (− 6.9)	23.9 (− 7.9)	< 0.001
Female (*n* = 10)	24.0	18.5 (5.5)	17.8 (− 6.2)	10.4 (13.6)	18.9 (− 5.1)	8.7 (− 15.3)	0.111
Perceptual evaluation—grade							
Overall (*n* = 61)	1.5	1.9 (0.41)	1.6 (0.11)	1.4 (− 0.11)	1.3 (− 0.20)	1.4 (− 0.05)	< 0.001
T1a (*n* = 29)	1.3	1.6 (0.32)	1.2 (− 0.09)	1.1 (− 0.17)	1.1 (− 0.19)	1.0 (− 0.29)	0.027
T1b (*n* = 19)	1.8	2.4 (0.69)	2.1 (0.34)	1.7 (− 0.08)	1.7 (− 0.03)	1.9 (− 0.15)	0.003
T2 (*n* = 13)	1.7	1.9 (0.23)	1.8 (0.08)	1.6 (− 0.07)	1.3 (− 0.39)	1.7 (− 0.01)	0.198
Male (*n* = 51)	1.5	2.0 (0.48)	1.6 (0.11)	1.4 (− 0.10)	1.4 (− 0.12)	1.5 (− 0.03)	< 0.001
Female (*n* = 10)	1.6	1.6 (0.01)	1.5 (− 0.12)	1.4 (− 0.22)	1.0 (− 0.58)	1.4 (− 0.25)	0.481

∆difference between preoperative and the indicated follow-up time

#### EORTC QLQ-C30

Patients showed good global health status preoperatively and the improvement in global health status over time was only borderline significant. It increased from 77 preoperatively to 81 at 2 years postoperatively (∆4, *p* = 0.047). However, this improvement did not qualify as clinically relevant. The results of the different functional scales showed that patients reported high levels of functioning. In all five scales, scores ranged from 88 to 97 points after 2 years. One of these scales—the emotional functioning scale—showed a significant and clinically relevant improvement (∆18; *p* < 0.001), compared to preoperative values. The results of the symptom scales showed that patients also reported high levels of functioning on all items during the 2-year follow-up. The most common complaints were fatigue, dyspnea, and insomnia; scores ranged between 9 and 15 points. Only the change in insomnia showed a significant and clinically relevant improvement compared to preoperative values (∆11; *p* < 0.025) (Table 3).

**Table 3:** Quality of life scores on the EORTC QLQ-C30

EORTC QLQ-C30 item	Preoperative Mean	6 weeks Mean	3 months Mean	6 months Mean	1-year Mean	2-years Mean	*p* value
Global health							
Global health status	77	81	83	81	81	81	0.047
Functional scales							
Physical functioning	95	95	94	94	93	94	0.190
Role functioning	95	96	94	94	95	97	0.635
Emotional functioning	72	85	85	87	88	90	< 0.001
Cognitive functioning	89	91	89	92	91	88	0.362
Social functioning	92	93	95	96	95	95	0.402
Symptom scales							
Fatigue	20	15	14	15	17	12	0.118
Nausea and vomiting	1	1	2	3	3	3	0.329
Pain	6	7	3	4	7	5	0.173
Dyspnea	17	15	18	20	19	15	0.509
Insomnia	20	15	12	13	11	9	0.025
Appetite loss	5	2	4	5	3	4	0.857
Constipation	5	3	3	3	2	3	0.678
Diarrhea	4	6	4	2	5	3	0.429
Financial difficulties	6	4	3	3	2	3	0.262

#### EORTC QLQ-HN35

The symptom scales showed low symptom scores at 2 years after treatment. As seen in the VHI questionnaire, gender had a significant effect on two items. Females had significantly lower mean scores than males on speech problems (10.6 points difference; *p* = 0.037) and sticky saliva (11.8 points difference; *p* = 0.039). At 2 years postoperatively, most complaints were about speech problems (male patients), sexuality, sticky saliva (male patients), coughing, and the use of painkillers; the scores for these items ranged between 11 and 17 points. Nevertheless, compared to preoperative values, both speech problems (∆24, *p* < 0.001) and coughing (∆15, *p* = 0.002) showed significant and clinically relevant improvements at 2 years. Pain also showed a significant improvement (∆4; *p* = 0.02), but this improvement did not qualify as clinically relevant. The improvement in speech problems among female patients was clinically relevant, but the change was not significant (∆13, *p* = 0.691) (Table 4).

**Table 4:** Quality of life scores on the EORTC QLQ-HN35

EORTC QLQ-HN35 item	Preoperative Mean	6 weeks Mean	3 months Mean	6 months Mean	1 year Mean	2 years Mean	*p* value
Symptom scales							
Pain	8	5	5	6	6	4	0.029
Swallowing	3	3	2	2	4	3	0.863
Senses problems	5	5	3	4	5	4	0.855
Speech problems (M)	38	31	20	18	13	14	< 0.001
Speech problems (F)	20	15	10	10	10	7	0.691
Trouble with social eating	2	2	2	2	2	2	0.970
Trouble with social contact	4	4	2	3	4	4	0.269
Less sexuality	14	15	17	15	18	17	0.905
Teeth	9	3	9	8	6	8	0.329
Opening mouth	3	2	2	2	5	2	0.220
Dry mouth	18	13	17	19	15	9	0.079
Sticky saliva (M)	15	16	12	10	15	13	0.363
Sticky saliva (F)	0	4	0	5	4	0	0.963
Coughing	28	21	21	22	16	13	0.002
Felt ill	10	7	5	5	8	8	0.459
Painkillers	13	8	10	7	12	11	0.333
Nutritional supplements	1	2	1	0	4	0	0.169
Feeding tube	0	0	0	1	0	0	0.451
Weight loss	9	7	1	5	4	9	0.037
Weight gain	5	19	19	23	17	7	< 0.001

*M* male, *F* female

### Perceptual evaluation

The grade fluctuated significantly over time, and at 2 years, the pre- and postoperative values were similar (*p* < 0.001). Initial deterioration was observed at 6 weeks. Thereafter, recovery was noted, and the grade stabilized between the 3- and 6-month time points. The tumor stage (T1a, T1b, and T2) had a significant impact on the grade (*p* = 0.001). Patients with T1a tumors had significantly better end scores than patients with T1b tumors (difference in means: 0.76, *p* < 0.001) and patients with T2 tumors (difference in means: 0.49, *p* = 0.031). We found no significant difference between patients with T1b and T2 tumors (difference in means: 0.27, *p* = 0.256). At 2 years, the grade declined compared to preoperative values only in patients with T1a tumors (∆0.29; Table 2). Male and female patients did not have a mean difference in score (difference in means: 0.120, *p* = 0.644), although male patients fluctuated significantly over time (*p* < 0.001), whereas female patients did not (*p* = 0.481).

## Discussion

This prospective study investigated QoL and voice outcome for 2 years after TLM (unilateral type III resection or bilateral type II resection) in patients with early glottic carcinoma (extended T1 and limited T2). Our results indicate good overall QoL with low symptom scores. The voice outcome data showed slightly elevated VHI and grade scores. The VHI showed most improvement within the first 6 months. Interestingly, the VHI was significantly affected by gender; at 2 years after treatment, females showed scores within the normal range (8.7 points) and males showed slightly elevated scores (23.9 points). The grade score for dysphonia declined initially after surgery and showed most of the improvement or recovery within the first 6 months. The grade was significantly affected, not by gender but by tumor stage. At 2 years, the grade scores were between 1.0 (T1a) and 1.9 (T1b), which indicates mild (T1a) to moderate (T1b–T2) dysphonia. The final scores showed improvement for T1a tumors, but no change for T1b and T2 tumors, compared to preoperative grade scores.

This study is one of the first to find a significant effect of gender on the VHI questionnaire, although we could not confirm this in the perceptual evaluation. Both men and women showed improvements over time, but only female patients achieved clinically relevant improvements. However, most likely due to the small sample size of female patients, this improvement did not reach statistical significance. The female scores fluctuated over time, with an outlier score at 1 year. Therefore, in future studies with small groups, we recommend studying scores over time, rather than only evaluating two different time points. On the other hand, the improvement in male patients was statistically significant, but not clinically relevant. In contrast to these results, the study by van Gogh et al. reported no association between the VHI scores and gender, either in patients with voice impairments or in the population with normal glottic function [23]. However, in another study on patients with a variety of laryngeal diseases, Karlsen et al. found a correlation (*r* = − 0.17, *p* < 0.05) between the VHI score and gender; female patients had lower scores than male patients [30]. This latter finding was consistent with our results, although no exact VHI scores were given in that study [30]. The difference in VHI improvement between males and females might be explained by the fact that women show postoperative fundamental frequencies within the normal female range, whereas male patients show postoperative fundamental frequencies that are higher than the normal male range (van Loon et al. [18]). Potentially, this characteristic could lead male patients to experience a larger change in their voice and therefore to be less satisfied. The potential impact of gender on voice outcome after TLM for glottic carcinoma must be confirmed in future studies in larger patient populations. Until then, these results should be interpreted with caution.

The finding that patients with T1a tumors had significantly better grade scores than patients with T1b and T2 tumors might be explained by the fact that a lower tumor stage requires a smaller volume resection of the vocal cords [31, 32]. During the first 6 weeks, a temporary deterioration in grade was observed for tumors in all stages, followed by an initial recovery at 3 months. Between the 3rd and 6th months, the grade further improved and stabilized. This pattern was consistent with results reported in previous studies [33, 34]. At 2 years, only patients with T1a tumors showed significant improvements, compared to preoperative values.

After 6 months, only small changes in both the VHI and grade scores were observed. Therefore, improvements achieved at 6 months were indicative of the states achieved at 1 and 2 years postoperatively. Furthermore, the grade evaluations, showing mild to moderate dysphonia in both males and females, indicated that the voice did not return to normal levels after 2 years. This finding could be explained by the destructive effect of surgery on the vibratory layers of the vocal cord and the development of fibrosis. Interestingly however, the VHI did return to normal values for females and was only slightly elevated for males. This discrepancy between the VHI score and grade evaluation implies that there is a difference between what the patients experience and how experts rate their voices. This lack of correlation between the VHI questionnaire and the perceptual evaluation has been shown in other studies [12, 14, 35, 36]. A study by van Loon et al. investigated the time trade off in patients with laryngeal cancer and concluded that none of the patients who were treated with TLM was prepared to trade off years to live in perfect health. This shows that the perceived side effects (e.g., dysphonia) by patients are not substantial and that patients are able to cope well with their limitations in daily life [37]. QoL is a multidimensional construct; thus, it is best measured with an instrument that reports on multiple domains of functionality and well-being. Among the most widely used questionnaires in head and neck cancer research are those developed by the EORTC (QLQ-C30 and QLQ-HN35). These questionnaires have been used in many studies on patients with laryngeal cancer. However, they have been used in only a few studies on patients with early glottic cancer [10, 12, 16, 38, 39]. Three of these studies compared QoL in patients with early glottic cancer that were treated with either radiotherapy or TLM [10, 38, 39]. Two previous studies focused exclusively on patients that underwent TLM [12, 16]. The study of Hsin et al. prospectively investigated 62 patients with early glottic cancer (Tis-T2) that underwent TLM (ELS types I–VI) [16]. They demonstrated an immediate decline in QoL scores in the first few months, which recovered to baseline after 6 months, and then improved at 12 months, compared with preoperative scores. That finding is in contrast with findings in our study, because our patients did not report an immediate deterioration in QoL scores postoperatively. This difference might be explained by the fact that the previous study treated 15 patients (24%) with type IV–VI resections [16]; in contrast, we only treated patients with unilateral type III and bilateral type II resections.

Items on the QLQ-30 and QLQ-HN35 questionnaires have previously shown little differences in scores between men and women [40]. However, laryngeal cancer is less common in women than in men; thus, demonstrating differences between the sexes can be challenging, due to the limited number of female patients. Several studies on either general populations or patients with laryngeal cancer have shown that women reported significantly worse QoL scores than men [41–44]. In our study, the data did not confirm this gender difference. On the contrary, we found that men reported significantly more problems of speech and sticky saliva than women did on the QLQ-HN35 items.

On both questionnaires (QLQ-C30 and QLQ-HN35), we observed slightly elevated values (12–17 points) for fatigue, dyspnea, speech (male patients), sticky saliva (male patients), coughing, and sexuality after 2 years of follow-up. Compared to normative data from the general Dutch population, QLQ-C30 items (fatigue and dyspnea) that were less than 10 points different from the reference group [44] were not considered clinically relevant. In the literature, no study has reported normative data for the QLQ-HN35 questionnaire; therefore, the other slightly elevated items (speech problems, sticky saliva, coughing, and sexuality) could not be compared to a reference. One multinational study analyzed data on 293 patients with laryngeal cancer (stages I–IV). Although that study tested the reliability and validity of the head and neck cancer module, it did not report normative data. They included patients that were newly diagnosed, had recurrent disease or were disease free (1–3 years after treatment), and were primarily treated with radiotherapy [24]. Compared to those results, our patients reported fewer problems. This discrepancy might be explained by the fact that they included larger tumors than those we included, and their patients were treated primarily with radiotherapy. In the future, it would be interesting to generate normative data for the QLQ-HN35 module to enable comparisons with healthy individuals.

The strengths of this study is the prospective design and the duration of the follow-up. Due to the long-term follow-up, we were able to show that the results at 6 months and 1 year, which are more common time frames for these types of study, are representative for the long term. Our results have therefore been useful to us in counselling patients who undergo these specific resections on what to expect—both in terms of end results and the time frame within which these are achieved. The study had some limitations. First, due to the longitudinal nature of our study and the inclusion of patients from three different hospitals, we could not avoid missing data, despite the prospective study design. In addition, we did not collect data on patient comorbidities and smoking after treatment. This could be of relevance and interesting for further research. Second, we did not collect data on speech therapy. However, all patients were instructed by the speech–language pathologist in vocal hygiene after TLM. No patient received speech therapy before 3 months after surgery. After that, speech therapy was administered on a case-by-case basis. We acknowledge that speech therapy can improve the voice results, and therefore we advocate the collection of data on speech therapy in future studies as suggested by Heijnen et al. [45]. Third, the sample size of female patients was small. Therefore, the significant effects of gender on the VHI and QLQ-HN35 questionnaire must be confirmed in future studies with lager sample size. Fourth, in the QLQ-HN35 we found a slightly elevated score in the item sticky saliva. Normally, you may expect elevated scores for sticky saliva after the treatment with radiotherapy and not after the treatment with TLM. However, no explanation could be given by the authors as to why sticky saliva showed elevated scores. Therefore, it would be interesting to generate normative data for this questionnaire.

## Conclusion

Based on our findings, we conclude that patients with extended T1 and limited T2 tumors treated with TLM (unilateral type III or bilateral type II) show good QoL with low symptom scores and slightly elevated voice outcome data, at 2 years after treatment. Most of the improvement is observed within 6 months, and this level of improvement provides a clear indication of the status at 1 and 2 years postoperatively. These findings are useful for guiding patients in clinical decision making.
